# The salt between the beans: a qualitative study of the role of auxiliary midwives in a hard-to-reach area of Myanmar

**DOI:** 10.1186/s12913-019-3958-8

**Published:** 2019-02-28

**Authors:** Kyu Kyu Than, Stanley Luchters, Khaing Nwe Tin, Thazin La, James Beeson, Alison Morgan

**Affiliations:** 1 Burnet Institute, 85 Commercial Road, Melbourne, Victoria 3004 Australia; 2 Department of Medicine, Royal Melbourne Hospital, University of Melbourne, Melbourne, Australia; 3 International Centre for Reproductive Health, Department of Obstetrics and Gynaecology, Faculty of Medicine and Health Sciences, Ghent University, Ghent, Belgium; 4 Department of Epidemiology and Preventive Medicine, and Central Clinical School, Faculty of Medicine Nursing and Health Science, Monash University, Melbourne, Australia; 5 Department of Public Health, Ministry of Health and Sports, Nay Pyi Daw, Myanmar; 6 Nossal Institute for Global Health, Melbourne School of Population and Global Health, University of Melbourne, Melbourne, Australia

**Keywords:** Auxiliary midwives, Health system, Health care provider, Community perspectives, Maternal and child health, Hard-to-reach areas, Qualitative inquiry, Myanmar

## Abstract

**Background:**

Auxiliary Midwives (AMWs) are unpaid volunteer health workers assisting qualified paid midwives in maternal and child health care mainly in hard-to-reach areas of Myanmar. This paper describes the relationship between AMWs and the health system in providing maternal and child services as perceived by the community, AMWs themselves and health care providers in one remote township of Myanmar.

**Method:**

A qualitative study was conducted in Ngape Township, Myanmar. A total of 15 focus group discussions with midwives, AMWs, community members and mothers were conducted. Ten key informant interviews were performed with national, district and township level health planners and implementers of maternal and child health services. Thematic analysis was done using the ATLAS.ti software.

**Results:**

AMWs occupy a unique position between the community and the health sector in the study township. The relationship and trust with the community is built upon prolonged presence providing health care, skill building and fulfilling community expectations. Health care providers’ expectations to provide only preventive care, health promotion and education and childbirth care are often exceeded in reality when emergencies occur in hard-to-reach areas. This challenge to handle emergency situations with no support and limited skills and training is considered as most difficult by the AMWs. This mismatch of service provision expectations by both the community and other health care providers has put AMWs in a position which they describe as being the “salt between the beans” an essential ingredient but often invisible between the beans.

**Conclusion:**

The trust and relationship developed by AMWs over four decades of community practice serving as the mediator role is an untapped resource that can facilitate future community-based maternal and child health interventions in Myanmar.

## Background

The re-emergence of the role of community health workers in maternal and newborn health has been an integral strategy to combat the human resource crisis in many low and middle income countries [1–3].The services they provide have reduced childhood undernutrition, expanded access to family-planning services and improved maternal and child health [3, 4]. Community health workers vary in their roles, titles, duration of training, means of remuneration and the level of acceptance and inclusion within different health systems [2]. In general, community health workers are people selected from the community to carry out specific tasks related to health care delivery within the community and usually receive some training related to these tasks. They usually have a basic level of schooling and do not have formal professional or paraprofessional training [2, 3, 5].

Myanmar has a large deficit in human resources for health, with just 1.33 health workers (doctors, nurses and midwives) per 1000 people [6]. At the rural community level in Myanmar, three main cadres of community-based birth attendants serve mothers and children: Midwives (MWs), traditional birth attendants (TBAs) and Auxiliary Midwives (AMWs). MWs are skill birth attendants stationed at rural sub-centres and are the frontline maternal and child health workers within the government health care system. As 70% of its population lives in rural areas and majority of birth in these areas occurred at home where skilled birth attendance is low. Births that are not attended by MWs are usually assisted by an AMW or a TBA in the rural hilly areas, and comprise around 50% of all home births [6–8].

### AMW in the context of Myanmar

AMWs are community members who live in hard to reach rural villages of Myanmar. They are recruited from their respective villages to serve as unpaid voluntary health workers and trained for six months (three months of theory and three months of practice) by the township health department using an AMW training manual. AMWs trainings have been ongoing since 1978 till now on an irregular basis depending upon the funding availability within the health system. AMW trainings are guided by the Maternal and Reproductive Division under the Ministry of Health and Sports. Although AMWs are trained by the health system, they are not part of the system general structure for remuneration [9]. The aim of training and deploying AMWs was to assign them in villages where there are no qualified paid MWs, and to replace the TBAs who are considered unskilled health workers. AMWs are selected from the communities based on selection criteria that include secondary level of education and work commitment to serve back to their own local community. Following the six month training period, they provide antenatal care, nutrition education, breast feeding information and support, counselling, and post-natal care to mothers and new-borns within their local area. They are also trained to conduct normal deliveries and identify risks and complications for timely referral. They do not receive any salary or incentives from the government health system, but often receive cash and kinds from the community for acknowledgement of their services [10, 11]. There are 30,799 AMWs and 13,529 MWs present in 64,134 villages of Myanmar at the end of 2015 [8].

Myanmar’s government has renewed interest in optimising the role of AMWs for rural health development, by expanding the role of existing AMWs. The World Health Organization (WHO) has also recommended a range of competencies and practices that can be implemented by AMWs [12]. In a previous study we examined the role of AMWs and the potential for task shifting essential maternal interventions (oral supplement distribution to pregnant women; administration of misoprostol to prevent postpartum haemorrhage and management of puerperal sepsis with oral antibiotics) [13]. Moreover, studies have demonstrated that implementing effective interventions successfully requires an understanding of the relationship and trust between health worker, community and the health system in which the intervention aims to take place [14, 15]. Also studies on AMWs in Myanmar reported that AMWs’ knowledge and skills are low and of concern [16–18] and training and supervision are also identified as weak and inadequate [17, 19]. Currently there is a lack of knowledge about the perceived value and expected roles of AMWs by the community and health care workers. In this study we aimed to describe the relationship between AMWs and the health system in providing maternal and child services as perceived by the community, AMWs themselves and other health care providers in one hard-to-reach area of Myanmar, to provide the contextual understanding for optimising their role.

## Method

The study was part of a larger study looking at optimizing the role of AMWs for task shifting maternal and child health interventions and the methods and qualitative questionnaire guide have been reported in two separate publications [13, 20]. The method in brief is as follows.

The qualitative inquiry was conducted in Ngape Township (an administrative subdivision of a district) in Myanmar. Ngape Township has an estimated population of 46,572 and has one 25 bedded township hospital with 12 medical staff, one 16 bedded station hospital with 5 medical staff and three rural health centres which provide out-patient care to the community with 4 basic health workers in each centre. There are 28 MWs, 99 AMWs and only 1 TBA in Ngape township serving a total of 105 villages.

The consolidated criteria for reporting qualitative research (COREQ) checklist was used to report the methods and findings of the study [21].

The research team consisted of one experienced qualitative researcher who conducted all interviews and focus group discussions, and four research assistants involved in note-taking and transcribing the audiotapes. The research team participated in a three-day training on the background and rationale of the study, its objectives, ethical considerations and on strengthening certain qualitative research techniques more specifically.

The topics covered in the interviews and focus group discussions (FGDs) in relation to the present paper were as follows (Table 1).

**Table 1:** Themes and sub themes explored in FGDs and Interviews

Themes	Emerging themes in the interview
**Perceptions of health care providers** (How do the health care providers perceive the role of AMWs in the health system)	Relationship and trust (relationship and trust between providers and AMWs in the health system)
	Expectations (role and performance expectation from AMWs by the system/providers)
**Perceptions of the community** (How do the mothers and the community perceive the role of AMWs in providing care for them)	Relationship and trust (relationship and trust between mothers, community and AMWs)
	Expectations (role and performance expectation from AMWs by mothers/community)
**Perceptions of being an AMW** (Self-reflection of being an AMW including positive and negative outlook and difficulties encountered)	Relationship and trust (relationship and trust between AMWs and mothers, community, MWs, TBAs and the township health people)
	Expectations (role and performance expectation towards AMWs by the community, MWs and township health people)

For the key informant interviews, at least two people from each of the category of government health system (township/region/central) were involved. A total of ten key informant interviews were conducted (three central/national level health planners, five district and township level health planners and implementers, and two from the 3 Millennium Development Goal (3MDG) fund who were involved in maternal and child health program implementation). The national level is the key decision-making level of the health system. District level health departments provide supervisory and technical support to the township level and guide the process of AMW recruitment, supervision, training and decision making towards AMW roles and tasks. Township level health departments manage the township health system which is the backbone of primary health care, provides comprehensive health services at the local level and is predominantly responsible for management of the AMW activities [7]. Moreover, fifteen FGDs with MWs, AMWs, community members and mothers of children under the age of three years were conducted in two hard-to-reach villages and two non-hard-to-reach villages (Table 2). Village selection was determined through discussion with the township health authorities and we decided that with the time, available resources and the limit of transportation we decided to go to 2 hard to reach and 2 non-hard to reach villages.

**Table 2:** Overview of study participants of the focus group discussions and in-depth interviews

Category	Number of FGDs	Total number of participants	Characteristics
Auxiliary Midwives (AMWs)	5	33	1. AMWs from hard-to-reach villages with service year more than 5 years 2. AMWs from hard-to-reach villages with mix service years 3. AMWs from non-hard-to reach villages with service year less than 5 year 4. AMWs from non-hard-to-reach villages with service year more than 5 years 5. AMWs from non-hard-to-reach village with mix of service years
Midwives (MWs)	2	15	One with MWs assigned in hard- to-reach areas and one with MWs assigned in non-hard-to-reach areas.
Mothers with children under three years of age	4	29	Two with mothers from hard-to- reach areas and two with mothers from non-hard-to-reach areas
Community members	4	36	Two with community members from hard-to-reach areas Two with community members from non-hard-to-reach areas
Key informants	–	10	Three national level, two district and three township level and two from Three MDG fund

The criteria for categorization of the hard-to-reach and non-hard-to-reach villages are based on the geographical hard-to-reach definition used by the 3MDG programme based on scores allocated for travelling time to the nearest facility, mode of travel, transport charges and roads affected by seasonal variation. A total of 12 points are given and villages scoring 0 to 3 are considered as non-hard-to-reach, while 4 and above as hard-to-reach villages. Each FGD consisted of between five and twelve participants. FGDs with MWs were conducted in the township hospital and all other FGDs in community gathering places of the respective villages and the office of an international non-government organization (NGO) in Ngape Township to ensure confidentiality and privacy. All the individual interviews were done in private places chosen by the interviewees, and were mostly carried out in the offices of interviewees.

All the FGDs were audio recorded with written informed consent from the participants. Three interviewees refused to be audio recorded; all other individual interviews (IDIs) were audio recorded. Note taking was done for all the IDIs and FGDs. All audio recordings were transcribed verbatim in Myanmar language from the digital recorders by the note takers and checked against field notes for consistency. The durations of the FGDs and IDIs ranged from 30 min to 90 min with an average duration of 50 min. Before the actual coding, all transcripts were read and reread by the principal author in Myanmar language and by the data coders. Data coding was done using the ATLAS.ti software. Two members of the research team were directly involved in coding the transcripts. Reliability coding was set at 80% agreement and the inter-coder reliability was found to be over 80%. The primary coding structure was developed using the primary topic domains (roles, responsibilities and perceptions of the function of AMWs) and sub themes were identified after a thorough familiarisation with the transcripts. Participant characteristics (key informants, MWs, AMWs, mothers and community members) were also considered during the coding. Before finalising the code structure, the two researchers who coded the transcripts reviewed the coding structure and agreed on the final version. Thematic analysis was done using both inductive and deductive approaches.

## Results

The AMWs’ unique position and their relationships within the community and the township health system were discussed from perspective of health care providers, community members, and AMWs themselves.

### Providers’ perception of AMWs

All health planners and MWs endorsed the critical role of AMWs in remote and hard-to-reach settings, and as the repository for important local community knowledge. AMWs are considered by the health care providers who participated in the study as a necessary cadre for the health system, especially in the hard-to-reach areas where government appointed health workers were not able to go.

*“As long as MWs are not fully appointed in villages, we will always need AMWs. For the remote areas, it is a wise decision to have the AMWs as a helping hand”* (District level key informant).

MWs, who are the immediate supervisors of AMWs, stated that building good relationships with AMWs was a necessity in reaching to the hard-to-reach villages. AMWs were considered as the best persons with local knowledge to help MWs conduct community health care activities. One MW who was assigned in the hard-to-reach village expressed how the AMW assisted her to carry out her daily activities.

*“We need good relationship with the AMWs because they are the local people who know the villagers and help us in our daily activities”* (MW from hard-to-reach).

*“We (MWs) are assigned to four to five villages. We cannot go to all the villages as some are very far and we think having AMWs in these far away villages is an asset for the community and the health system”* (MW from hard-to-reach village).

Rapport was built by acknowledging AMWs’ activities. MWs believed that this was important in ensuring the ongoing engagement and commitment of the AMWs given their volunteer status.

*“We persuade them. You are volunteers and you do good things for your village. We have to tell them their good points”* (MW from non-hard-to-reach).

*“Sometime, we had to request the AMW to gather pregnant women and children in their homes for immunization and health talks….because the villages are so far away we had to sleep and eat in their houses…..we cannot give anything back...”* (MW from hard-to-reach).

### Providers’ expectations of AMWs role

Health care providers expressed varying opinions when describing the scope of AMW services. Many stated that they expect AMWs to do preventive and promotive care but they also mentioned that AMWs need to know how to perform life-saving interventions before referring the patient to the hospital. One MW who had experienced difficulties in transferring a mother with obstetric complications to hospital, stated that,

*“There will be benefit if they know how to put an IV* [intravenous injection] *drip line. I think we should teach AMWs in hilly villages. So, there is someone who can take care of mothers if something happens in an emergency”* (MW from non-hard-to-reach).

*“We do need to teach them first line emergency care management as they often bring the patient in a life-threatening condition such as severe bleeding without any treatment from faraway places….if the patient can be given at least first line management….lives would be saved”* (MW from hard-to-reach).

At the same time, there are contrasting opinions about the AMWs’ responsibility. One key informant expressed his concern that AMWs were practicing beyond their competencies without fear of the consequences.

*“Their main role is to assist the MWs in her routine activities like immunization and assist at normal deliveries and refer risk cases. However, in some of the areas they are giving injection and delivering high risk cases”* (Township key informant).

*“It’s really hard to know what they do in their villages as we cannot see them with our own eyes…. Supervision is limited and they sometime go beyond their capacity”* (Township key informant).

National and District level key informants mentioned the role of Township Medical Officers (TMOs) in imparting and maintaining expectations and norms for AMWs. The TMO is the responsible person for making decisions at township level. All volunteer recruitment and training is under the supervision of the TMO and the decision and support given by the TMO is of utmost importance for the AMWs.

*“It depends on the relationship and leadership of the Township Medical Officer (TMO). If the TMO is strong and can control the MWs and the AMWs, the township is under control. If the TMO is weak and cannot make firm decisions the township becomes hectic and this creates problems”* (District key informant).

### Community’ perceptions of AMWs

Community members perceived the AMW as a valuable member of the community. Many of the mothers and community members spoke about the good relationship they had with AMWs who have been in their villages all their lives. Notably there were no practicing TBAs in villages where there were experienced AMWs. Trust and confidence was related to prolonged presence as well as the perception of AMW competencies and skills.

*“Everyone in the village relies on her* [AMW] *as she is very skilful and understanding…she is like a family member, she stays with us all night ….when my son was born she did not sleep at all...”* (Mother from non-hard-to-reach).

*“She knows us all, in our villages we see her as a valuable and trustable person, she is understanding, helpful and takes care of us like her own family member, we don’t worry and think about money when we go to her. We just give her rice, vegetables…. whatever we have…”* (Community member from non-hard-to reach).

However, villagers from the hard-to-reach village in which there was a newly appointed AMW (trained in 2015) expressed their concern about her capability in assisting at childbirth; comparing her to the traditional birth attendants and the old experienced AMW who had served them for a long time.

*“…the previous AMW could do everything, assisting at the birth of children and giving injections and she was very skilful, now she has moved to Thailand, so we have to give birth with the traditional birth attendant….she (new AMW) is not experienced yet….just finished training”* (Community member from hard-to-reach).

They also mentioned that time will be needed to build the skills of young AMWs.

*“She will need about three years to get the skills and confidence”* (Community member from hard-to-reach).

Because of the prolonged presence and the services provided, some of the older community members in the interviews were confused between an AMW and a MW. They trust and rely on the AMW describing her as their own skilled person who takes care of their health.

*“We have our own Sayarma* [General name used for a MW in Myanmar Language] *for so long and she is so skilful, we trust her and everyone relies on her ….just recently there is a government appointed one in the nearby village…but we like and trust our own better*” (community member from non-hard-to-reach).

Community members and mothers in particular considered that AMWs have good relationships with the MWs and whenever health activities are carried out in the village, the AMWs were considered as a primary communicator and spokesperson for the village.

*“She is the main health person for the village… she helps the MWs in immunization and she also can connect her [MW] when we need them [MWs]”* (mother from hard-to-reach).

*“We always see her helping MWs and they both get along well. MWs sometimes go to meetings in the township and we have to rely on our AMW”* (community member from non- hard-to-reach).

### Community expectations of AMW role

In one of the hard to reach villages, there is a traditional birth attendant who still conducts deliveries as well as a young newly trained AMW. The main difference expressed by the community between an AMW and a Traditional birth attendant was that an AMW would be able to provide injectable medications. AMWs who have been serving the community and fulfilling their demand was considered as a skilful person.

*“They* [AMW and TBA] *both can help child birth…but AMWs can provide injections and are more educated”* (community member from hard-to-reach).

*“She is very skilful……she treats our illness……she gives very good injections and she explains to us about our condition in a language we understand.”* (Mother from non-hard-to -reach)

In this village, the newly trained AMW who did not provide any injections was perceived by the villagers as unsatisfying. They mentioned that they wanted a MW instead of an AMW who could provide injections and do everything necessary for the mothers and their community.

*“She is inexperienced as she is young, so we give birth with a TBA and when we ask for injections she said she doesn’t do it”* (mother from hard-to-reach).

*“The previous AMW is a bit older and has more experience and does everything childbirth, injections and very good at treating fever… if she gives medicine fever goes away”* (mother from hard-to-reach).

Although the government has trained AMWs to assist MWs in maternal and child health activities, many of the community members in the hard-to-reach areas expected more from the AMWs than they were able to offer. They expected an AMW to be a skilled provider who could provide all health care services equivalent to a MW. Many community members want AMWs to make less referral and to give more injections. In spite of the restriction on injection practice by AMWs from the government, many of the mothers and the community expressed their demand for injections without being aware of the restrictions.

*“She went to training for so long and she is still reluctant to give the injection. We sent her to be like a MW, who could do all”* (community member from hard-to-reach).

Many of the community members highlighted the cost related to service provision and AMWs are considered as persons who provide affordable care compared to the MWs.

*“She [AMWs] knows our situation in and out and we can pay her when we have it and sometime we just show our appreciation by giving rice and food but we can’t do that to MWs, we need money to pay for her [MW] service”* (community member from non-hard-to-reach).

### AMWs perceptions of the health care providers and the community

#### Relationship and trust with the providers and community

AMWs in the study mentioned that relationship and trust are built within the community with dedication and time. Many of the older AMWs said that they devoted all their life to this work in which trust was built. Younger AMWs also mentioned that they had to be involved in many of the community activities to gain the trust of the community.

*“For an AMW, we must have 3 rules…. we must have ‘saydana’* [literally meaning compassion]*, ‘wathana’* [literally meaning interest] *and ‘anitna’* [literally meaning endurance]*”* (AMW from non-hard-to-reach).

However, a few AMWs mentioned that in spite of all the effort in caring for the community the only reward they get back is blame for being a lesser skilled volunteer in the health system.

*“Sometimes it is so hard to persuade them* [patient and family] *to go to the hospital especially in critical conditions and to reach there* [hospital] *is so difficult, and hospital staff start scolding us saying ‘why did you bring a dead body, you should have brought her alive’.”* (AMW from hard-to-reach).

The relationship with the MWs was mostly expressed as positive and attendance at birth with MWs was perceived as helpful and encouraging by most of the AMWs. However, they also mentioned about the difficulty faced by some families in terms of cost for two providers at birth.

*“They only want to call one, we told them not to worry about money as both of us delivered her. We do not ask the fee from the poor ones, we just told them to pay for the motorbike* [transport] *cost of Sayarma* [General name used for a MW in Myanmar Language] *and for us we just told them not to worry”* (AMW non-hard-to-reach).

#### AMWs response to expectations of others

Although some of the AMWs in the study mentioned that they treated mothers and community members for fever and minor ailments, reluctance was observed when describing the provision of drugs and injections. AMWs in the studied township had mixed feelings regarding what they can do and are allowed to do for the community with the voluntary status of their role. Providing curative care in emergency situation was articulated as demand driven.

*“The child had a head injury and it was bleeding…..I told the father to go to the hospital, ‘how can I go to the hospital, I have no money’…he* [the father] *just sat there in my home… not moving….the child is bleeding and I know I need to stop it ….so I stitched his* [the child] *head…. and gave him* [the child] *Anti Tetanus Toxoid injection……….”* (An experienced AMW from hard-to-reach).

*“some of the community members think we are paid by the government, although we explain again and again that we are volunteers and do not get any money, they still insist us on some of the tasks that we are not allowed to do like injection”* (An AMW from hard-to-reach).

More experienced AMWs in the study mentioned that in the 1990s when they undertook training, they were expected to learn and provide curative care to the community as there was no skilled provider in or near by their villages.

*“…she* [MW] *taught me how do give injections and told me that if she is not around I can give….so I give injections”* (experienced AMW in hard-to-reach).

#### Gatekeeper role of the township medical officers

AMWs in the studied township had mixed feelings regarding their responsibilities and what they are allowed to do was shaped by the township level authority. Many of the AMWs in the FGD mentioned about the different level of permissions and restrictions rendered by different TMOs concerning their role as AMWs. They were enthusiastic in telling stories about many of the TMOs who have trained and taught them to be who they are today in serving their own village.

*“It was safe with him* [TMO A]*. We were trained on how to give treatment and injections. He said he will take responsibility. Then we were not scared to treat before sending the patients to the hospital”* (AMW from hard-to-reach).

Comparisons were made between TMOs that participants considered to be strict, and TMOs that participant considered to be cooperative, which influenced their perception of their own role and performance.

*“During his time* [TMO C]*, I was very depressed because he scolded me and told me to quit as an AMW because he found out that I gave an injection to the poor villager with fever. He was very strict”* (AMW from non-hard-to-reach).

AMWs also expressed their frustration at being in the middle between the health care system and the community in providing curative care. Many participants spoke about the lack of protection and support provided by the township health system. One stated,

*“I feel sad sometimes, we get badly scolded if something went wrong, but for the MW they are protected well. When they need us they say we are together with them in the system, when they don’t we are volunteers. So we are like salt between the beans. My family told me to quit the job but I have been doing this for more than 10 years and I can’t let go”* (Experienced AMW hard-to-reach).

## Discussion

This study suggests that trust and relationship building with the community was a function of the length of service of individual AMWs, their perceived skill, affordability, responsiveness to community demands for curative care, and how AMW interact with other health care providers. These findings are similar to other studies in low and middle income countries, where building trusting relationships involves not only the competency of the individual health worker but is also a function of other contextual factors including the outcome of services provided, responsiveness to need, and strong engagement with the community and the health system [15, 22, 23].

Older AMWs with long service and experience are more trusted and accepted for their skills compared to the younger AMWs who are newly trained and reluctant to provide curative care because of the rules set by the health authorities. It takes time, and in our study this was many years, and experience to build strong relationships and gain the respect of the people that the AMWs want to serve. A recent study looking at factors influencing productivity and willingness to serve the community by AMWs in Myanmar showed that older AMWs were more confident in taking care of the patient and were more likely to stay in service for more than 5 years [11]. This presents challenges for new AMWs, who, regardless of skill, lack this history. However a study by Calnan and Rowe argued that trusting relationships depended not only upon the length of service but also upon the outcome of the service provided [24]. Consequently the challenge is to build the skills and confidence in the newly trained AMWs. One potential strategy to better address the community expectations is to change AMW training curricula, adding selected curative practices that have been successfully provided by AMWs in the past.

Our study also highlights the importance of defining community expectations: either AMWs need to be better skilled and supported to provide selected curative practices, or more effort is required to ensure communities understand the clear boundaries of the AMW roles, to avoid unreasonable treatment demands being made on poorly equipped AMWs.

AMWs are caught between the community expectation that they be skilled sufficiently to provide emergency care including injections, and the health care providers’ mixed messages, where AMWs are allowed to provide curative care only for emergency conditions. Moreover, AMWs are given little, if any, guidance, and receive no support or training for when or what emergency care they can provide. A study done in Mozambique, also reported that unrealistic expectations by the community was a function of less accessible health facilities and challenging geography [1]. Our findings also showed that management for mothers during emergency conditions was frightening and daunting for AMWs, not only due to their limited skills, but also due to the distance from health care infrastructure. Curtale et al., reported that although the community health workers in Nepal tried to respond to the community expectations during emergency conditions, their fear and frustration were mentioned due to inadequate skill and training [25]. To ensure that AMWs are supported for their risk-taking clinical interventions, clear policy for AMWs’ role along with skills-based practical training is highly recommended in order to respond to emergency situations in hard-to-reach areas.

Another factor for consideration is the voluntary status of the AMW despite the requirements of considerable training. Since there is no compensation for their services from the formal health system, responding to community demands may mean building trust as well as doing a livelihood to earn an income. Although providing monetary incentives to community health workers is still a debatable issue, a systematic review done by Kok et al., 2014 stated that compensation and career development opportunities often enhance community health workers’ motivation along with community trust and respect [22]. Therefore, compensation for service along with career development pathways needs to be considered for AMWs.

There is evidence that lay maternal health workers with similar educational backgrounds to AMWs in Myanmar, after two years’ working experience, have been able to deliver basic emergency obstetric care [26] in internally displaced communities in Eastern Burma/Myanmar through skill based training and team building. However, this project was resource intensive and the sustainability of the approach may require extensive skilled based training curriculum with highly skilled trainers, sufficient supplies and necessary equipment, and a monthly stipend to support the health workers [26]. Other feasible interventions to prevent maternal mortality and morbidity, such as the distribution of misoprostol for prevention of postpartum haemorrhage, monitoring of blood pressure of mothers and treating them with low dose aspirin and providing antibiotics for postpartum sepsis have been shown to be of effective and have recently been proposed as interventions that could be used by AMWs according to the WHO task shifting guidelines recommendations [20, 27]. Both financial and infrastructure resources are needed to build the skills of AMWs in responding to emergency situations including effective referral.

The findings from the present study also highlight the additional importance of the gate keeper role of Township Medical Officers in shaping the role and performance of AMWs at the implementation level. Any efforts to skill up AMWs need to take into account the township level leadership and governance along with policy level decentralization. Thus to impose the value of the salt (AMWs) between the beans (community and the health system) a strong policy commitment along with supervision, skills-based training and compensation need to be taken into account.

### Strengths and limitations of the study

Limitations of this study include that it was not able to assess the wider national and district level factors that may contribute to the relationships of AMWs, and the quality of care provided by AMWs at the township level was not assessed. In addition, the findings may have been affected by social acceptability bias of AMWs at the community level as in some hard-to-reach villages they are the only health care provider present. However, the research team attempted to mitigate this by involving different range of participants from both hard-to-reach and non-hard-to-reach villages. Despite these limitations, the study results bring attention to policy makers and a wider research community that AMWs have positive trusting relationships with not only the community but are also embedded within the health system of Myanmar as an essential cadre for maternal and child health in rural areas.

## Conclusion

Being the valuable “salt” between the health system and the community, AMWs need to be protected through the provision of enabling policy environment, adequate supervision, skills based training, compensation and indemnity to provide health services in hard to reach areas.

## Abbreviations

3MDG: The Three Millennium Development Goal Fund
AMW: Auxiliary midwife
FGD: Focus group discussion
IDI: Individual depth interview
KII: Key informant interview
MW: Midwife
NGO: Non-Government Organization
TBA: Traditional birth attendant
TMO: Township Medical Officer
WHO: World Health Organization
